# Evolution of frontline treatment of diffuse large B-cell lymphoma: a brief review and recent update

**DOI:** 10.12688/f1000research.8790.1

**Published:** 2016-08-08

**Authors:** Jung Yong Hong, Cheolwon Suh, Won Seog Kim

**Affiliations:** 1 Department of Oncology, Asan Medical Centre, University of Ulsan, Seoul, 05505, Korea, South; 2 Division of Hematology-Oncology, Department of Medicine, Samsung Medical Centre, Sungkyunkwan University, Seoul, 135-710, Korea, South

## Abstract

Various strategies have been implemented to improve the outcomes of diffuse large B-cell lymphoma (DLBCL). In recent years, remarkable advances have been achieved, based on the discovery of cell-of-origin in DLBCL and on more effective targeted agents. This commentary will summarize recent updates on the evolution of frontline therapies for DLBCL, focusing on the upcoming promising frontline chemotherapy platforms and on activated B-cell subtype DLBCL and double-hit DLBCL.

## Introduction

Diffuse large B-cell lymphoma (DLBCL) is the most common subtype of non-Hodgkin lymphoma. Therapeutic advances have been achieved in DLBCL with the addition of the anti-CD20 monoclonal antibody rituximab to cyclophosphamide, doxorubicin, vincristine, and prednisone (R-CHOP) ^1, 2^. Despite this, about one-third of patients with DLBCL do not achieve durable remission and develop relapsed/refractory disease ^3^. Various strategies have been implemented to improve the outcomes of DLBCL. In this review, we summarize recent updates on the evolution of frontline therapies for DLBCL, focusing on the development of more effective chemotherapy platforms and on subtype-specific therapy.

## Toward more effective chemotherapy platforms beyond R-CHOP

### R-ACVBP (rituximab, doxorubicin, cyclophosphamide, vindesine, bleomycin, and prednisone)

The Groupe d'Etude des Lymphomes de l'Adulte investigated increased dose-intensity approaches to improve on R-CHOP. They showed that intensified immunochemotherapy with R-ACVBP significantly improved the survival of patients ^4^. The primary endpoint was event-free survival (EFS). After a median follow-up of 44 months, 3-year estimates of EFS were 81% (95% confidence interval [CI] 75–86) in the R-ACVBP group and 67% (95% CI 59–73) in the R-CHOP group (hazard ratio [HR] 0.56, 95% CI 0.38–0.83, P=0.0035). Three-year overall survival (OS) (92% [87–95] versus 84% [77–89], HR 0.44 [0.28–0.81], P=0.0071) was also increased in the R-ACVBP group. The R-ACVBP group showed significantly increased but manageable grade 3–4 hematological toxicities, with a proportion of febrile neutropenia of 38% (75/196) compared with 9% (16/183) in the R-CHOP group ^4^. However, we should interpret these positive results for R-ACVBP with caution for the following reasons. First, some of the drugs in the regimen are not available in all countries and testing of substitute drugs or modifications would be needed in real-world practice. Second, the inclusion criteria were limited to patients 18–59 years old and to patients with an age-adjusted international prognostic index (IPI) of 1. These inclusion criteria are not really applicable for the majority of patients with DLBCL. R-ACVBP was the first intensified multidrug regimen to improve on R-CHOP as a first-line treatment for DLBCL in a randomized phase III trial and R-ACVBP should be strongly considered in a substantial proportion of fit patients with curable DLBCL. However, the drawbacks mentioned above restrict its potential as a universal first-line platform for DLBCL treatment.

### DA-EPOCH-R (dose-adjusted etoposide, prednisone, vincristine, cyclophosphamide, and doxorubicin with rituximab)

Several international groups investigated another increased dose-intensity regimen, DA-EPOCH-R ^5– 7^. The National Cancer Institute group showed that the progression-free survival (PFS) and OS at 5 years were 79% and 80%, respectively, and suggested that DA-EPOCH-R was a promising first-line treatment for DLBCL, especially in low- and intermediate-IPI groups ^5^. The Cancer and Leukemia Group B reported that time to progression (TTP) and OS rates were 81% and 84% at 5-years, respectively, with a median follow-up of 62 months and showed that DA-EPOCH-R provided highly durable remissions in both germinal center B-cell (GCB) and non-GCB subtypes ^6^. Most recently, the Spanish Programa Español de Tratamientos en Hematología group reported 10-year follow-up data showing a good long-term outcome and a tolerable toxicity profile of DA-EPOCH-R in high-risk large B-cell lymphoma patients (IPI higher than 2 or age-adjusted IPI higher than 1) ^7^. Based on these promising results and favorable toxicity profiles, a randomized phase III study comparing DA-EPOCH-R with R-CHOP (NCT00118209) has completed patient recruitment. The primary endpoint is EFS and results are pending.

### Optimization of rituximab administration

Further intensification of chemotherapy may not be feasible in older patients, so further intensification of rituximab appears to be an attractive strategy because of its wide therapeutic window. The Deutsche Studiengruppe Hochmaligne Non-Hodgkin Lymphome (DSHNHL) group assumed that early dose densification of rituximab in combination with CHOP-14 might be beneficial and tested a schedule of administration of 375 mg/m ^2^ rituximab on days 0, 1, 4, 8, 15, 22, 29, 43, 57, 71, 85, and 99, combined with six cycles of CHOP-14, comparing this with eight 2-week applications of rituximab plus the same chemotherapy schedule (DENSE-R-CHOP-14 trial). Unfortunately, although dose-dense rituximab achieved higher rituximab serum levels, it was not more effective than eight 2-week applications, even though there were minor improvements in outcomes for male patients with a poor prognosis ^8^. The DSHNHL group also tested whether prolonged rituximab exposure might improve the efficacy of R-CHOP. They administered 375 mg/m ^2^ rituximab on days -4, 0, 10, 29, 57, 99, 155, and 239, together with six cycles of R-CHOP-14 (SMARTE-R-CHOP-14 trial). Interestingly, they reported that compared with eight 2-week applications, extended rituximab exposure significantly improved the outcomes of older poor-prognosis patients without increasing toxicities ^9^. The superiority of the SMARTE-R approach will be tested in an ongoing phase III trial using an optimized schedule of rituximab and liposomal vincristine (OPTIMAL>60 trial, NCT01478542) and the primary endpoint of the study is PFS. In previous trials of DLBCL, elderly male patients demonstrated significantly lower rituximab serum levels and worse outcomes than those in elderly female patients. The randomized phase II SEXIE-R-CHOP-14 trial was designed to test the administration of increased doses of rituximab in elderly male patients with DLBCL ^10^. Increasing rituximab dose from 375 mg/m ^2^ to 500 mg/m ^2^ eliminated the increased risk of elderly male patients, showing that PFS rates after a period of 3 years were 74% in males and 68% in females (P=0.396) and the 3-year OS rates were 82% and 72%, respectively (P=0.111) ^10^. In terms of maintenance therapy, a recent large multicenter phase III trial (NHL13 trial) including 662 DLBCL patients showed that rituximab maintenance therapy during the first remission does not significantly alter the outcome for patients, showing that 3-year estimates of EFS (the primary endpoint) at 3 years is 80% for rituximab maintenance versus 77% for observation (HR 0.79 [0.57–1.08], P=0.1433) and OS also remains unchanged (92% versus 90%) ^11^. However, 10% EFS and PFS benefit of rituximab maintenance in subgroup analysis of male patients appears to be worthy of further investigation.

### Obinutuzumab-CHOP

Obinutuzumab (GA101), a new, humanized, monoclonal type II anti-CD20 antibody modified by glycoengineering, demonstrated responses in single-arm studies of patients with relapsed/refractory non-Hodgkin lymphoma ^12, 13^. Obinutuzumab had an acceptable and manageable safety profile. A phase II trial (GATHER) of obinutuzumab plus CHOP (G-CHOP) as first-line chemotherapy for untreated CD20+ DLBCL suggested that G-CHOP could be a safe and effective regimen and showed the dose intensity of CHOP was maintained throughout treatment ^14^. The study demonstrated that the ORR was 83% (complete response [CR] 55% [44/80], partial response [PR] 28% [22/80]) and PFS/OS were not fully evaluated due to a short follow-up period. Now, a randomized phase III study (GOYA, NCT01287741) comparing G-CHOP with R-CHOP as first-line treatment for DLBCL has completed patient recruitment and results are pending. The primary endpoint of the study is PFS and the results are being watched with keen interest to see whether G-CHOP can replace R-CHOP as a new universal first-line platform for DLBCL treatment.

## Toward subtype-specific therapy, the first step to precision medicine

### Cell-of-origin in DLBCL

DLBCL is a heterogeneous disease with a variety of clinical presentations. Gene expression profiling (GEP) has classified DLBCL into different molecular cell-of-origin (COO) subtypes: GCB, activated B-cell (ABC), and primary mediastinal B-cell (PMB) lymphoma and a minority of unclassified DLBCL ^15^. DLBCL COO subtypes have distinct pathobiology and show striking differences in clinical outcomes, with the ABC subtype being associated with the worst outcomes ^16, 17^. BCL2-driven malignant transformation is one of the principal pathophysiological mechanisms of GCB-subtype DLBCL, and constitutive activation of the NF-κB pathway is the hallmark of ABC-subtype DLBCL ^15– 17^.

The gold standard method to assign COO relies on the detection of intact RNA using fresh-frozen tissue (e.g. Lymphochip microarray or Affymetrix array). These are the most accurate methods, but their high cost and long turnaround time, and the limitations in the availability of patient samples (fresh-frozen tissue only), make these platforms impractical for routine use in practice. Considerable efforts have been made to approximate the results of this gold-standard method using practical technology platforms ^18^.

***Immunohistochemistry-based assays.*** Hans *et al*. suggested using an immunohistochemistry (IHC)-based COO assignment algorithm comprising CD10, BCL6, and MUM1 but showed only 79% concordance with cDNA microarray ^19^. Since then, a number of other IHC-based COO assignment algorithms have been developed, named after their proposers Choi, Tally, Visco, Colomo, and Muris ^18^. However, their concordance rate is unsatisfactory, and Gutierrez *et al*. reported disappointing results, which showed that the proportion of cases misclassified by IHC-based approaches ranges from 30% to 60% and that none of the IHC-based algorithms detected prognostic differences between GCB and non-GCB subtypes of DLBCL ^20^. Until now, IHC-based approaches were widely used in the United States and worldwide. However, these approaches have their limitations, as mentioned above.

***GEP-based assays using formalin-fixed paraffin-embedded tissue.*** Recently, technologies have been developed that allow GEP using the fragmented RNA derived from formalin-fixed paraffin-embedded tissue (FFPET). The major two platforms are cDNA-mediated annealing, selection, extension, and ligation (DASL) and NanoString (Lymph2Cx assay) ^21– 23^. Recent reports have shown robust results with the GEP-based technique using the Lymph2Cx assay, with a high concordance (>95%) between independent laboratories and significant prognostic value according COO ^23, 24^. Lymph2Cx is also the first GEP-based assay to demonstrate consistent interlaboratory performance. Considering its rapid turnaround time, accuracy, and the convenience of using FFPET, it is hoped that diverse GEP-based assays using FFPET will provide more uniform and consistent assignment of COO in DLBCL, such as Lymph2Cx assay, quantitative nuclease protection assay, ICEPlex ^®^ system, massive parallel quantitative RT-PCR, and reverse transcriptase multiplex ligation-dependent probe amplification assay ^25– 28^.

### ABC-subtype DLBCL

ABC-subtype DLBCL is characterized by chronic active BCR signaling, constitutive MYD88 signaling, and subsequent NF-κB pathway, AKT/mTOR pathway, and interferon pathway activation ^15– 17, 29^. The ABC subtype is associated with the worst outcome among COO subtypes of DLBCL when treated with standard R-CHOP chemotherapy. Therefore, the majority of clinical trials of novel agents in combination with R-CHOP as front-line treatment of DLBCL that are currently underway are specifically targeting the ABC subtype of DLBCL.

***Ibrutinib plus R-CHOP.*** Constitutively activated signaling through BCR and its associated protein tyrosine kinases (such as Bruton’s tyrosine kinase [BTK]) play a crucial role in the development and survival of malignant B-cells, including having involvement in the pathogenesis of the ABC subtype of DLBCL ^29^. A phase 1/2 trial of ibrutinib (BTK inhibitor) has shown selective activity against ABC-subtype DLBCL ^30^. A phase Ib trial of ibrutinib plus R-CHOP showed that ibrutinib and R-CHOP did not affect each other’s pharmacokinetics, that ibrutinib is well tolerated when added to R-CHOP, and that all 18 patients with DLBCL who received the recommended phase 2 dose had an overall response ^31^. Based on these data, a randomized, double-blind, phase III trial (PHOENIX, NCT01855750) comparing ibrutinib plus R-CHOP with placebo plus R-CHOP in newly diagnosed non-GCB-subtype DLBCL has completed patient recruitment. The primary endpoint of the study is EFS and the results are pending.

***Lenalidomide plus R-CHOP.*** Lenalidomide is an orally active immunomodulatory drug that has direct antineoplastic activity plus indirect effects mediated through multiple types of immune cells found in the tumor microenvironment, including B-, T-, natural killer, and dendritic cells ^32^. The antineoplastic effects of lenalidomide in ABC-subtype DLBCL were associated with direct targeting of IRF-4, leading to downregulation of NF-κB pathway activity and augmentation of the interferon pathway ^32, 33^. Recently, the results of two phase II trials of lenalidomide plus R-CHOP in DLBCL were published. An Italian group (REAL07 trial) reported an overall response rate (ORR) of 92% (CR 86%, PR 6%) and 2-year PFS and OS rates of 80% (95% CI 64–89) and 92% (95% CI 79–97), respectively ^34^. The Mayo Clinic also demonstrated that the ORR was 98% (59 of 60), with 80% (48 of 60) achieving CR, and EFS and OS rates at 24 months were 59% (95% CI 48–74) and 78% (95% CI 68–90), respectively. Notably, the study revealed that lenalidomide combined with R-CHOP overcame the negative prognostic impact of a non-GCB phenotype, showing no difference in 24-month PFS or OS for lenalidomide plus R-CHOP patients on the basis of non-GCB and GCB subtype (60% versus 59% [P=0.83] and 83% versus 75% [P=0.61] at 2 years) ^35^. Based on these data, a randomized, double-blind, phase III trial (ROBUST, NCT02285062) comparing lenalidomide plus R-CHOP versus placebo plus R-CHOP in newly diagnosed ABC-subtype DLBCL defined by central GEP assay (Lymph2Cx) is open and is actively recruiting. The primary endpoint of the ROBUST trial is EFS.

***Bortezomib plus R-CHOP.*** Proteasome inhibitors play a key role in the suppression of the transcription factor NF-κB, a downstream component of the BCR signaling pathway that is constitutively activated in ABC-subtype DLBCL ^18, 36^. A landmark study showed that bortezomib sensitized and enhanced the activity of chemotherapy in ABC-subtype DLBCL ^37^. The study showed that bortezomib combined with DA-EPOCH in relapse/refractory DLBCL yielded a CR of 18% and a PR of 16% and demonstrated a significantly higher response rate (83% versus 13%, P<0.001) and median OS (10.8 versus 3.4 months, P=0.003) in ABC DLBCL compared to GCB DLBCL, respectively ^37^. However, two recent phase II trials (LYM-2034 trial and PYRAMID trial) comparing bortezomib plus R-CAP or bortezomib plus R-CHOP versus R-CHOP in newly diagnosed non-GCB-subtype DLBCL have failed to show improved efficacy in terms of ORR and PFS ^38, 39^. Offner *et al*. demonstrated that there were no significant differences between bortezomib plus R-CAP and R-CHOP in CR rate (64% versus 66%, OR 0.91, P=0.80), ORR (93% versus 99%, OR 0.21, P=0.11), PFS (HR 1.12, P=0.76), or OS (HR 0.89, P=0.75) ^38^. Leonardo *et al*. also showed that there were no significant differences between bortezomib plus R-CHOP and R-CHOP in 2-year estimates of PFS (82% versus 78%, P=0.61) and OS (93% versus 88%, P=0.76) ^39^. However, further clinical trials may not be precluded by these results: in the LYM-2034 trial, the chemotherapy dose intensity was suboptimal in the bortezomib plus R-CAP arm, and in the PYRAMID trial, the outcomes of the R-CHOP arm were better than expected considering the non-GCB subtype. In addition, both trials are performed based on IHC-based, but not GEP-based, COO assignment. These factors should be considered when we interpret the results of the LYM-2034 and PYRAMID trials. Preliminary results of a randomized, double-blind, phase III trial (REMoDL-B trial) comparing bortezomib plus R-CHOP versus R-CHOP in newly diagnosed ABC-subtype DLBCL defined by central GEP assay (DASL) showed no difference in PFS of ABC-subtype and GCB-subtype patients (2-year PFS 71%) ^40^. This study indicates that bortezomib may help to overcome the poor prognosis of ABC-subtype DLBCL. The REMoDL-B trial is still awaiting the 30-month follow-up and the final results are pending.

### Double-hit DLBCL

Double-hit lymphomas (DHLs) are a heterogeneous group of mature B-cell lymphomas that harbor rearrangements of MYC and BCL2. However, MYC/BCL6 DHLs or MYC/BCL2/BCL6 “triple-hit” lymphomas can also occur ^41, 42^. The majority of DHL cases fall into the categories of DLBCL or B-cell lymphoma, unclassifiable, with features intermediate between DLBCL and Burkitt lymphoma. Virtually all double-hit DLBCLs are GCB subtype. Double-hit DLBCL showed a very aggressive clinical course and had a dismal prognosis when treated with standard R-CHOP treatment ^43^. Notably, DHLs or triple-hit lymphomas are classified in the new category of ‘high-grade B-cell lymphoma (HGBL), with rearrangements of MYC and BCL2 and/or BCL6’ in the 2016 updated WHO classification ^44^. “Double-expresser” lymphomas are defined based on IHC stains with MYC and BCL2 staining in more than a specified proportion of tumor cells. Most double-expresser DLBCLs are found within the ABC subtype of DLBCL. Double-expresser DLBCL also has an inferior outcome compared with classical DLBCL and has an intermediate outcome between double-hit DLBCL and classical DLBCL ^43, 45– 47^. However, we should interpret these results with caution owing to the retrospective nature of these data.

***DA-EPOCH-R.*** Currently, the optimal frontline chemotherapy in double-hit DLBCL is not well defined. Previous retrospective analysis has suggested that DA-EPOCH-R could be a viable option in double-hit DLBCL because it improved ORR and PFS compared with R-CHOP ^48, 49^. Recently, Howlett *et al*. reported a meta-analysis comparing R-CHOP, DA-EPOCH-R, and dose-intensive (DI) regimens (R-Hyper-CVAD and R-CODOX-M/IVAC). They showed that the median PFS for the R-CHOP (n=180), R-EPOCH (n=91), and DI (n=123) groups were 12.1, 22.2, and 18.9 months, respectively. First-line treatment with R-EPOCH significantly reduced the risk of progression compared with R-CHOP treatment ^50^. Promising preliminary results have been reported for a phase II study (NCT01092182) of DA-EPOCH-R in MYC-rearranged aggressive B-cell lymphomas, which showed that the PFS, TTP, and OS were 79%, 86%, and 77%, respectively, at a median follow-up of 14 months ^51^. Further analysis of these data with longer follow-up is planned.

***Novel agents.*** The two major strategies for novel agents in DHLs are 1) modulating the transcription of MYC, BCL2, or BCL6 and 2) targeting MYC, BCL2, or BCL6 proteins. For modulation of the transcription of MYC, BCL2, or BCL6, epigenetics-based treatment with diverse BET bromodomain inhibitors may show promise (BAY1238097 [NCT02369029], CPI-0610 [NCT01949883], OTX015 [NCT01713582], and JQ1) ^43^. For targeting the proteins, BCL2 inhibitors (navitoclax and venetoclax) and a MYC-targeting aurora A kinase inhibitor (alisertib) show promise and warrant further evaluation ^43, 52, 53^.

## Conclusions

We are at the threshold of an era of precision therapy for DLBCL, of which subtype-specific therapy would be the first step. Ongoing trials may in the near future change the current chemotherapy backbone R-CHOP and provide strategies for using combinations of novel agents with the chemotherapy backbone according to the molecular subtypes of DLBCL defined by GEP assay.

## Abbreviations

ABC, activated B-cell; BTK, Bruton’s tyrosine kinase; CI, confidence interval; COO, cell-of-origin; CR, complete response; DA-EPOCH-R, dose-adjusted etoposide, prednisone, vincristine, cyclophosphamide, and doxorubicin with rituximab; DASL, cDNA-mediated annealing, selection, extension, and ligation; DHL, double-hit lymphoma; DI, dose intensive; DLBCL, diffuse large B-cell lymphoma; DSHNHL, Deutsche Studiengruppe Hochmaligne Non-Hodgkin Lymphome; EFS, event-free survival; FFPET, formalin-fixed paraffin-embedded tissue; GCB, germinal center B-cell; GEP, gene expression profiling; G-CHOP, obinutuzumab, cyclophosphamide, doxorubicin, vincristine, and prednisone; HR, hazard ratio; IHC, immunohistochemistry; IPI, international prognostic index; ORR, overall response rate; OS, overall survival; PFS, progression-free survival; PMB, primary mediastinal B-cell; PR, partial response; R-ACVBP, rituximab, doxorubicin, cyclophosphamide, vindesine, bleomycin, and prednisone; R-CHOP, rituximab, cyclophosphamide, doxorubicin, vincristine, and prednisone; TTP, time to progression
